# Publisher Correction: Insights into the mechanism of phospholipid hydrolysis by plant non-specific phospholipase C

**DOI:** 10.1038/s41467-023-36155-2

**Published:** 2023-01-30

**Authors:** Ruyi Fan, Fen Zhao, Zhou Gong, Yanke Chen, Bao Yang, Chen Zhou, Jie Zhang, Zhangmeng Du, Xuemin Wang, Ping Yin, Liang Guo, Zhu Liu

**Affiliations:** 1 National Key Laboratory of Crop Genetic Improvement, Hubei Hongshan Laboratory, Huazhong Agricultural University, Wuhan, 430070 China; 2 Shenzhen Branch, Guangdong Laboratory for Lingnan Modern Agriculture, Genome Analysis Laboratory of the Ministry of Agriculture, Agricultural Genomics Institute at Shenzhen, Chinese Academy of Agricultural Sciences, Shenzhen, 518124 China; 3 Innovation Academy for Precision Measurement Science and Technology, Chinese Academy of Sciences, Wuhan, 430071 China; 4 Department of Biology, University of Missouri, St. Louis, MO 63121 USA; 5 Donald Danforth Plant Science Center, St. Louis, MO 63132 USA

**Keywords:** Enzyme mechanisms, Plant signalling

Correction to: *Nature Communications* 10.1038/s41467-023-35915-4, published online 12 January 2023

The original HTML version of this Article was updated shortly after publication because the previous HTML version linked to an incorrect Transparent Peer Review file.

