# Supplementary material for: Insights into the mechanism of phospholipid hydrolysis by plant non-specific phospholipase C
Source: Nat Commun. 2023 Jan 12;14:194. doi: 10.1038/s41467-023-35915-4 (PMC9837106; doi:10.1038/s41467-023-35915-4)
Supplement: Supplementary file 1 — Supplementary Information [file 41467_2023_35915_MOESM1_ESM.pdf]

Supplementary Information

Insights into the mechanism of phospholipid hydrolysis by plant non-specific phospholipase C

Ruyi Fan, Fen Zhao, Zhou Gong *et al*.

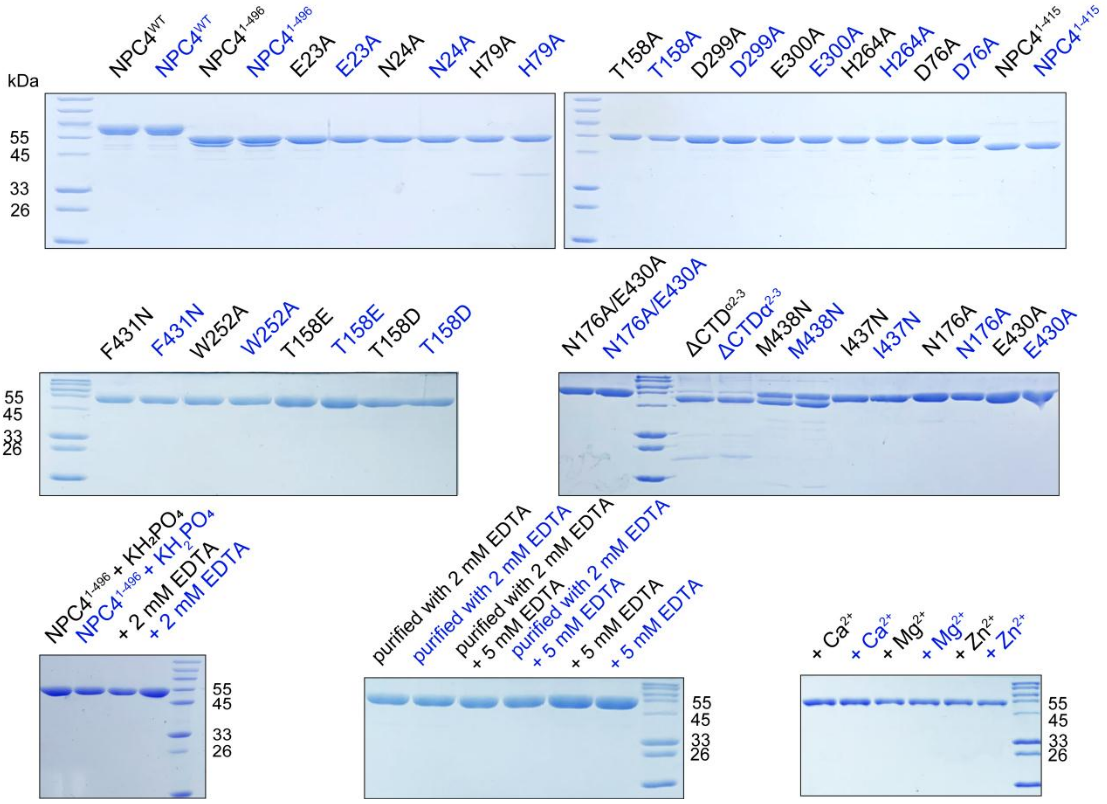

Supplementary Figure 1. Protein samples used for the activity assay in this study. Samples before (black) and after (blue) performing the substrate-hydrolysis reaction in the activity assay were examined using SDS-PAGE. The protein changed little after substrate-hydrolysis reaction, indicating that they were stable and no degradation happened in the activity assay. Each experiment was repeated independently at least three times with similar results. Source data are provided as a Source Data file.

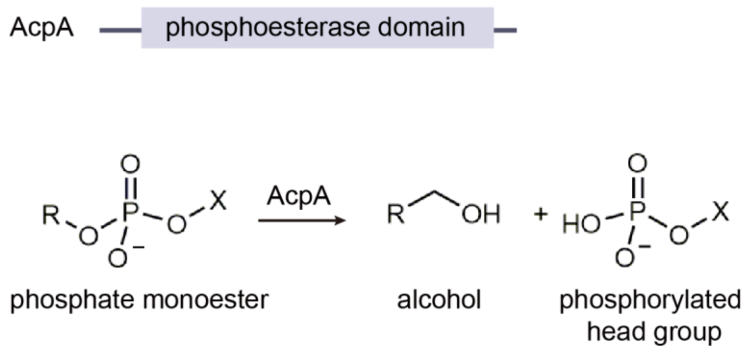

Supplementary Figure 2. Schematic depiction of AcpA and the chemical reaction catalyzed by AcpA. AcpA harbors only a phosphoesterase domain, lacking the C-terminal domain observed in NPC4.

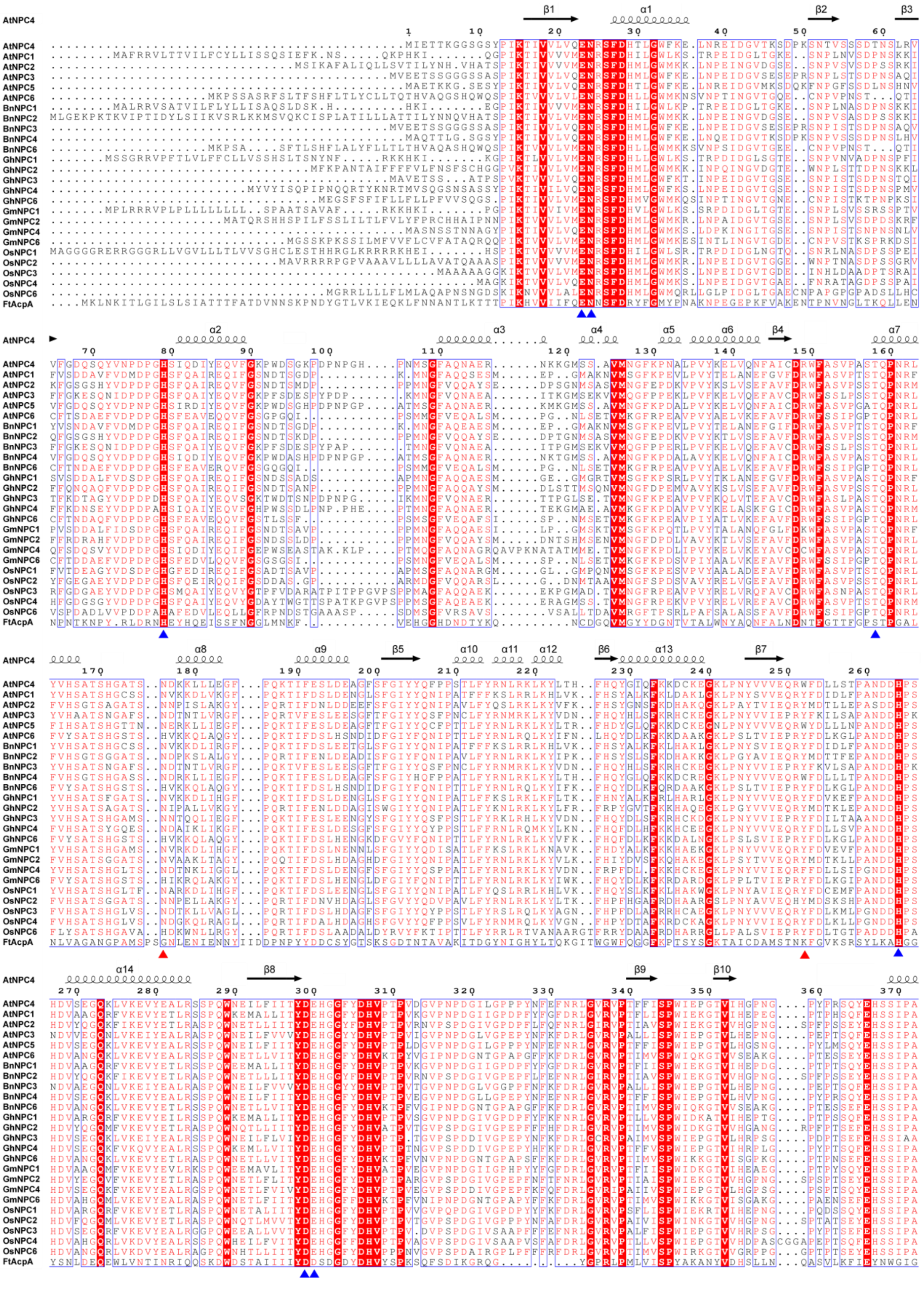
AtNPC4
β1
α1
β2
β3
α2
α3
α4
α5
α6
β4
α7
α8
α9
β5
α10
α11
α12
β6
α13
β7
α14
β8
β9
β10
AtNPC1
AtNPC2
AtNPC3
AtNPC5
AtNPC6
BnNPC1
BnNPC2
BnNPC3
BnNPC4
BnNPC6
GhNPC1
GhNPC2
GhNPC3
GhNPC4
GhNPC6
GmNPC1
GmNPC2
GmNPC4
GmNPC6
OsNPC1
OsNPC2
OsNPC3
OsNPC4
OsNPC6
FtAcpA

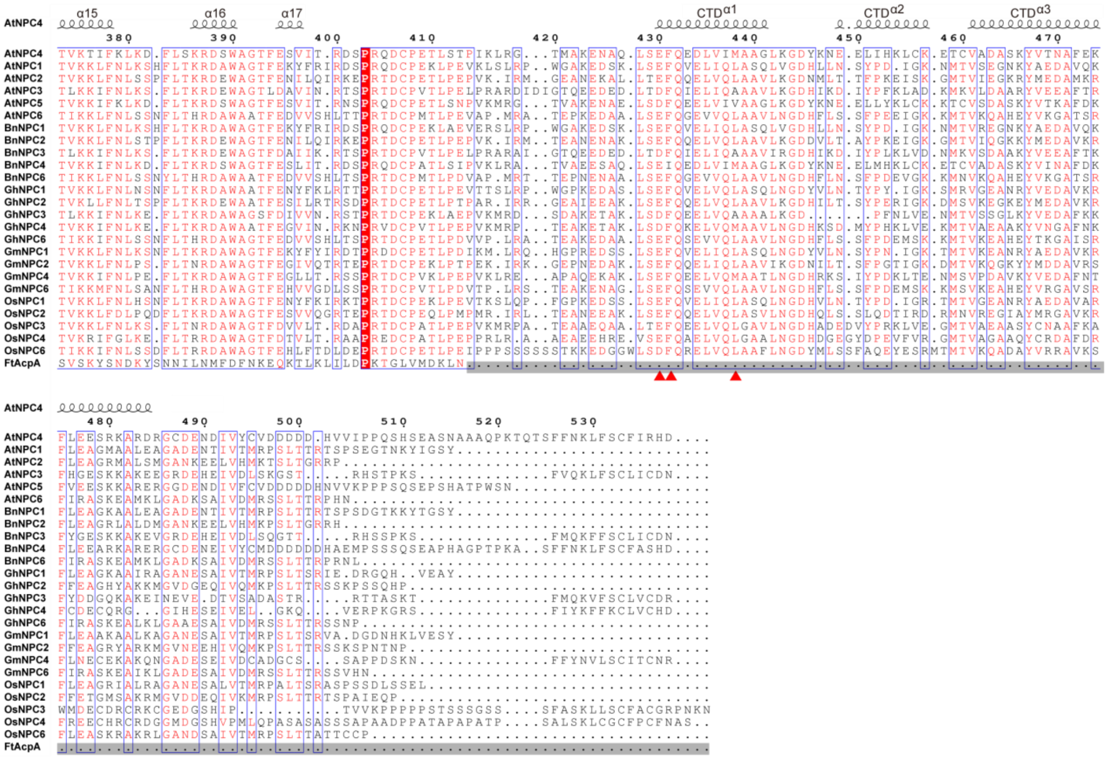

Supplementary Figure 3. Sequence alignment. The sequence of Arabidopsis NPC4 is aligned with other homologs. Structure-based alignment was performed by ESPript[1]. The sequence identity is indicated by white letters against a red background, and the sequence of a similarity over 90% is indicated by red letters. The secondary elements of AtNPC4 are labeled at the top of the alignment. Residues constituting the catalytic pocket and responsible for CTD$^{\alpha1}$-PD interaction are indicated with blue and red triangles, respectively. Gray bar highlights AcpA lacking the C-terminal domain that is observed in NPCs.

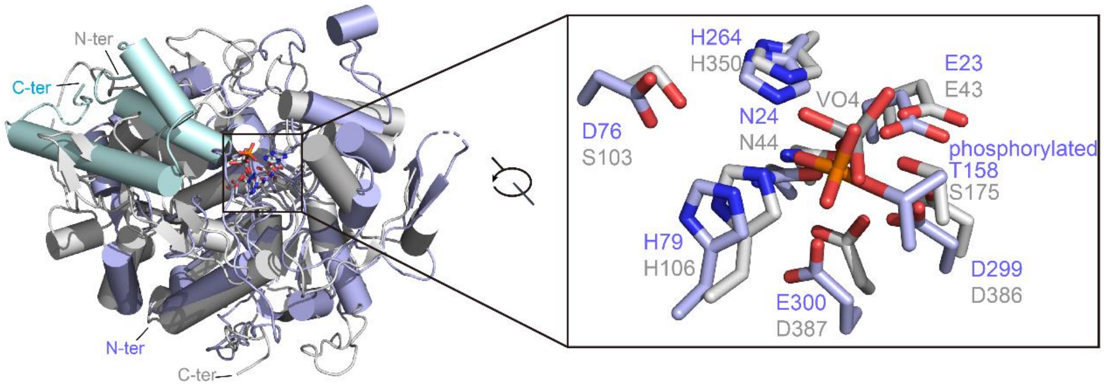

Supplementary Figure 4. Structure comparison between NPC4 and AcpA (gray cartoon, 2D1G.PDB). The PD domain of NPC4$^{1\text{-}496}$ (residues 16-398) is superposed to AcpA, with an overall RMSD of 3.27 Å. Residues constituting the active site are zoomed-in and shown in stick representation. The T158-linked phosphate in NPC4 and the orthovanadate inhibitor (VO_4) in AcpA are shown in sticks representation.

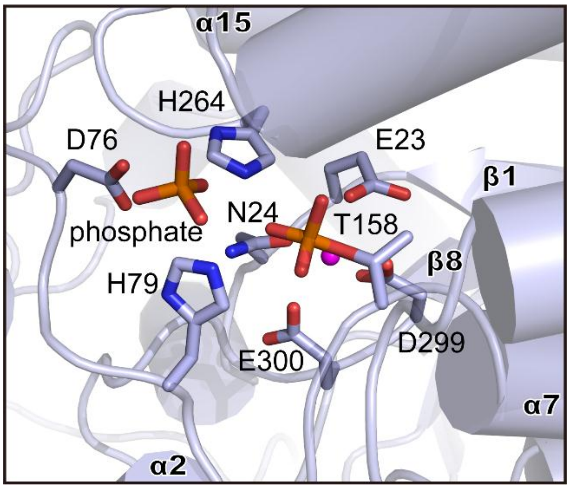

Supplementary Figure 5. Active site of NPC4$^{1\text{-}496}$. The observed molecules of phosphate and T158-linked phosphate are shown in stick representation. The metal ion is shown as magenta sphere.

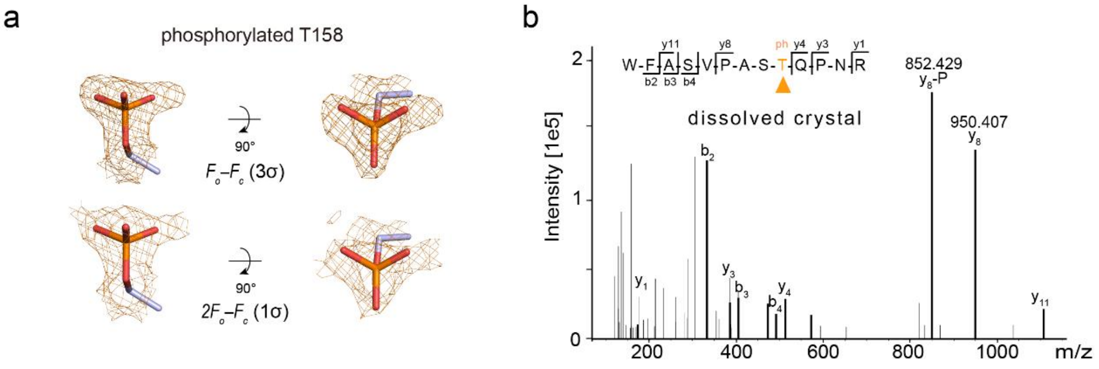

Supplementary Figure 6. **a**, The F_o - F_c electron density map (orange mesh and contoured at 3σ) and 2F_o - F_c electron density map (contoured at 1σ) of phosphorylated T158. Phosphothreonine is shown as stick, with atoms colored in wheat (carbon), red (oxygen) and orange (phosphorous), respectively. **b**, Mass spectrometry characterization for the phosphorylation of dissolved NPC4$^{1\text{-}496}$ crystal.

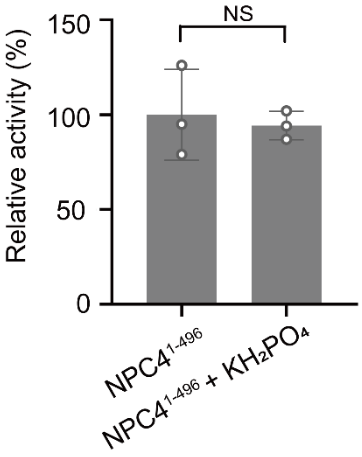

Supplementary Figure 7. Activity assay. Assay was performed for NPC4$^{1\text{-}496}$ in the absence or in the presence of 50 mM KH_2PO_4, a concentration of KH_2PO_4 that was used for crystallization. The data from three independent measurements are averaged, and the error indicates SD. Statistical analysis: unpaired two-tailed *t*-tests. NS, not significant. Source data are provided as a Source Data file.

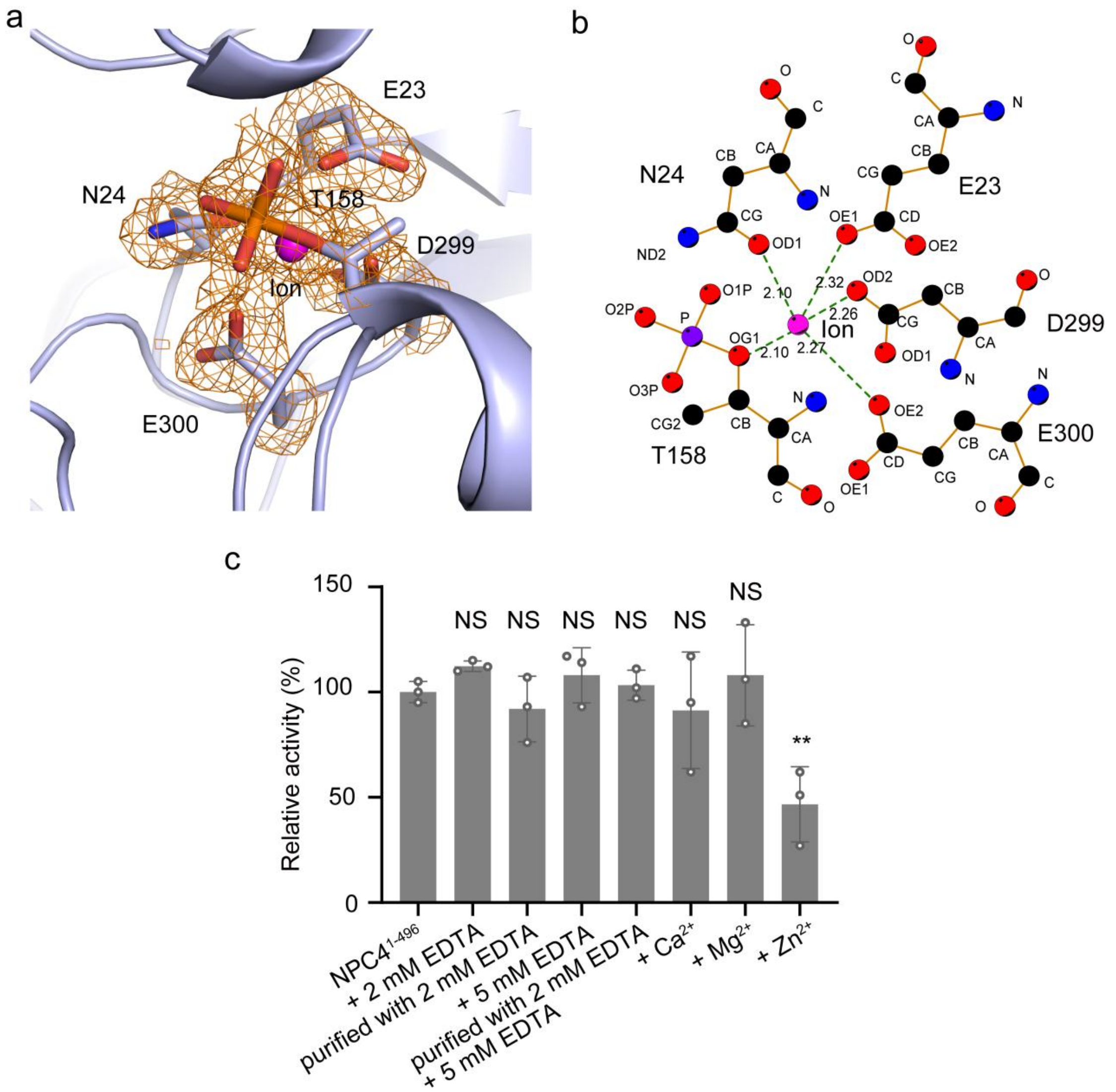

Supplementary Figure 8. a, The 2*Fo-Fc* electron density maps (orange mesh and contoured at 1σ) of metal ion and coordinating residues. **b**, Two-dimensional presentation of the metal ion and coordinating residues. **c**, Activity assay. Relative enzyme activity of NPC4$^{1-496}$ in the presence of each indicated additive is referenced to NPC4$^{1-496}$ without them. + 2 mM EDTA, 2 mM EDTA was added into the activity assay buffer; + 5 mM EDTA, 5 mM EDTA was added into the activity assay buffer; purified with 2 mM EDTA, NPC4$^{1-496}$ was purified in the presence of 2 mM EDTA and used for the activity assay; purified with 2 mM EDTA + 5 mM EDTA, NPC4$^{1-496}$ was purified in the presence of 2 mM EDTA and 5 mM extra EDTA was further added for measuring the enzyme activity; + Ca^{2+}, 1 mM $CaCl_2$ was added into the activity assay buffer; + Mg^{2+}, 1 mM $MgCl_2$ was added into the activity assay buffer; + Zn^{2+}, 1 mM $ZnCl_2$ was added into the activity assay buffer. The data from three independent measurements are averaged, and the error indicates SD. Statistical analysis: unpaired two-tailed *t*-tests. NS, not significant. **$p < 0.01$. p (+ Zn^{2+}) = 0.0076. Source data are provided as a Source Data file.

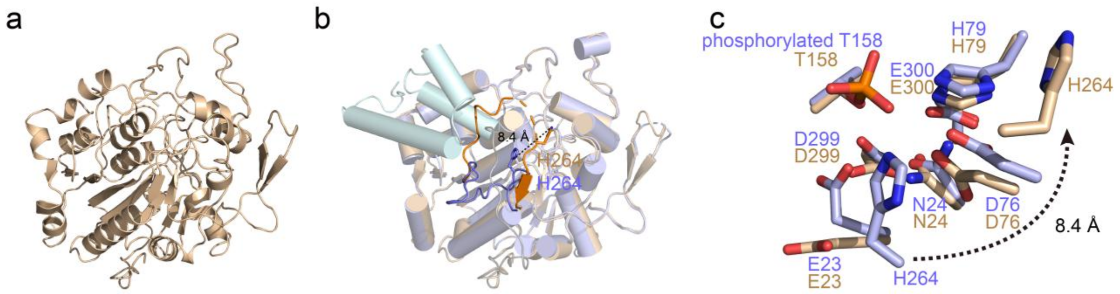

Supplementary Figure 9. a, Structure of NPC4$^{1\text{-}415}$. **b**, Structure comparison between NPC4$^{1\text{-}496}$ and NPC4$^{1\text{-}415}$. NPC4$^{1\text{-}496}$ is colored in the same scheme in Fig. 1d in main text. NPC4$^{1\text{-}415}$ is colored in orange. The loop (including residues 250-268) of NPC4$^{1\text{-}496}$ and NPC4$^{1\text{-}415}$ are colored in lightblue and orange, respectively. H264 is shown in stick representation. Residues of the active site are zoomed-in **c** and shown in stick representation. Black arrows denote local conformational changes.

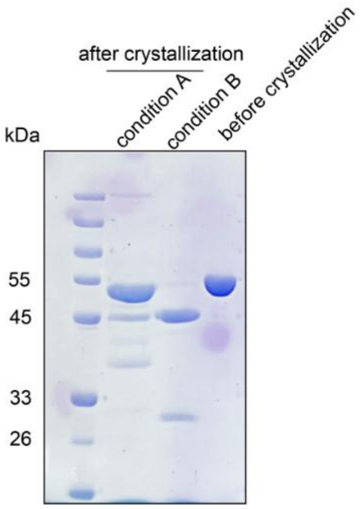

Supplementary Figure 10. Protein changes after crystallization under two conditions (see Methods for detail). Crystals matured under condition A and condition B were separately collected and subjected to SDS-PAGE analysis. Compared to the protein before performing crystallization, the protein crystallized under condition B appeared to be partially degraded. Condition A enabled us to determine the structure of NPC4$^{1\text{-}496}$, and condition B enabled us to determine the structure of NPC4$^{1\text{-}415}$. This analysis of crystals was performed once. Source data is provided as a Source Data file.

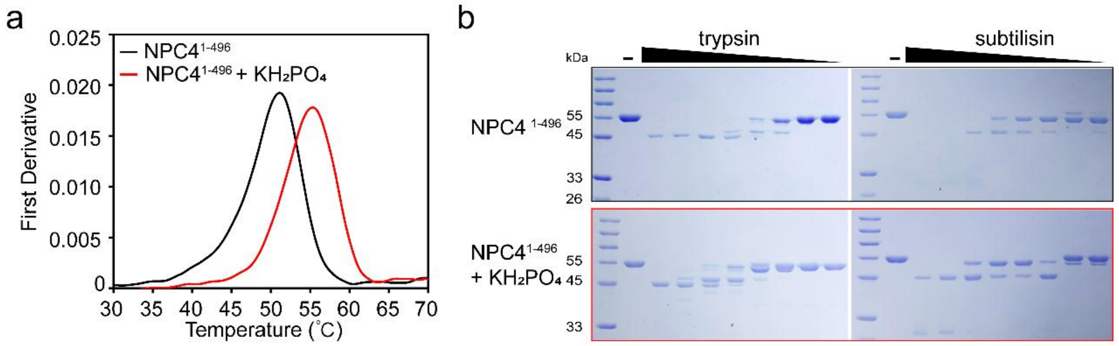

Supplementary Figure 11. KH_2PO_4 enhances the stability of NPC4$^{1-496}$. **a**, Different scanning fluorimetry (DSF) analysis. The melting temperature (Tm) of NPC4$^{1-496}$ is 51.1 ℃, and increases to 55.3 ℃ in the presence of 50 mM KH_2PO_4. Source data are provided as a Source Data file. **b**, Limited proteolysis experiments. NPC4$^{1-496}$ was treated with trypsin or subtilisin protease in the presence or absence of 50 mM KH_2PO_4, respectively. Results showed that NPC4$^{1-496}$ is more tolerant to these proteases in the presence of KH_2PO_4. Each experiment was repeated independently at least three times with similar results. Source data are provided as a Source Data file.

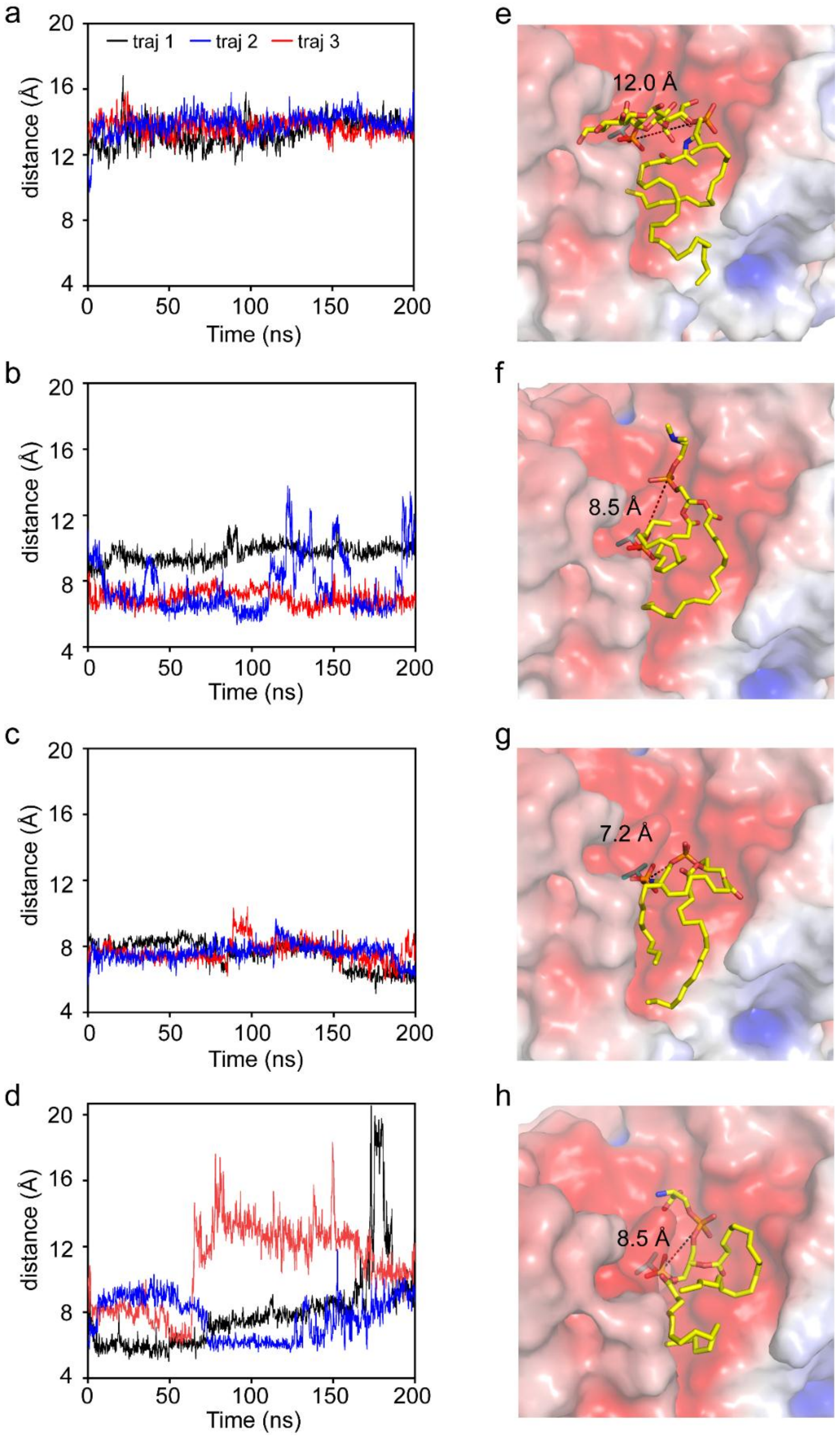

Supplementary Figure 12. a-d, Fluctuation of the distance between the nucleophilic atom of T158 and the phosphorus atom of each substrate during the simulations. Distance fluctuations were resolved from simulations of Figure 4e-l in main text. **e-h**, The distance between the nucleophilic atom of T158 and the phosphorus atom of substrate in the each docked NPC4$^{1\text{-}496}$-substrate model. Illustrations are presented in the same scheme in Fig. 4a-d in main text, with zoomed-in binding cleft.

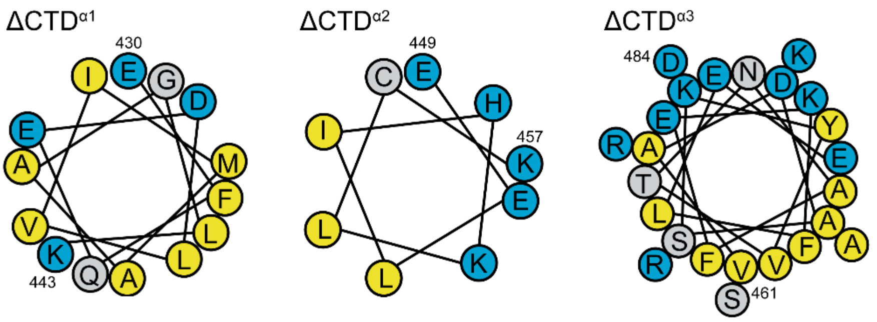

Supplementary Figure 13. Helical wheel diagram of the CTD 3 α-helices. CTD^{α1} includes residues 430-443, CTD^{α2} includes residues 449-457, CTD^{α3} includes residues 461-484. Hydrophobic residues, yellow; basic / acid residues, blue; uncharged polar residues, gray. The wheel drawing was analyzed using HeliQuest[2]

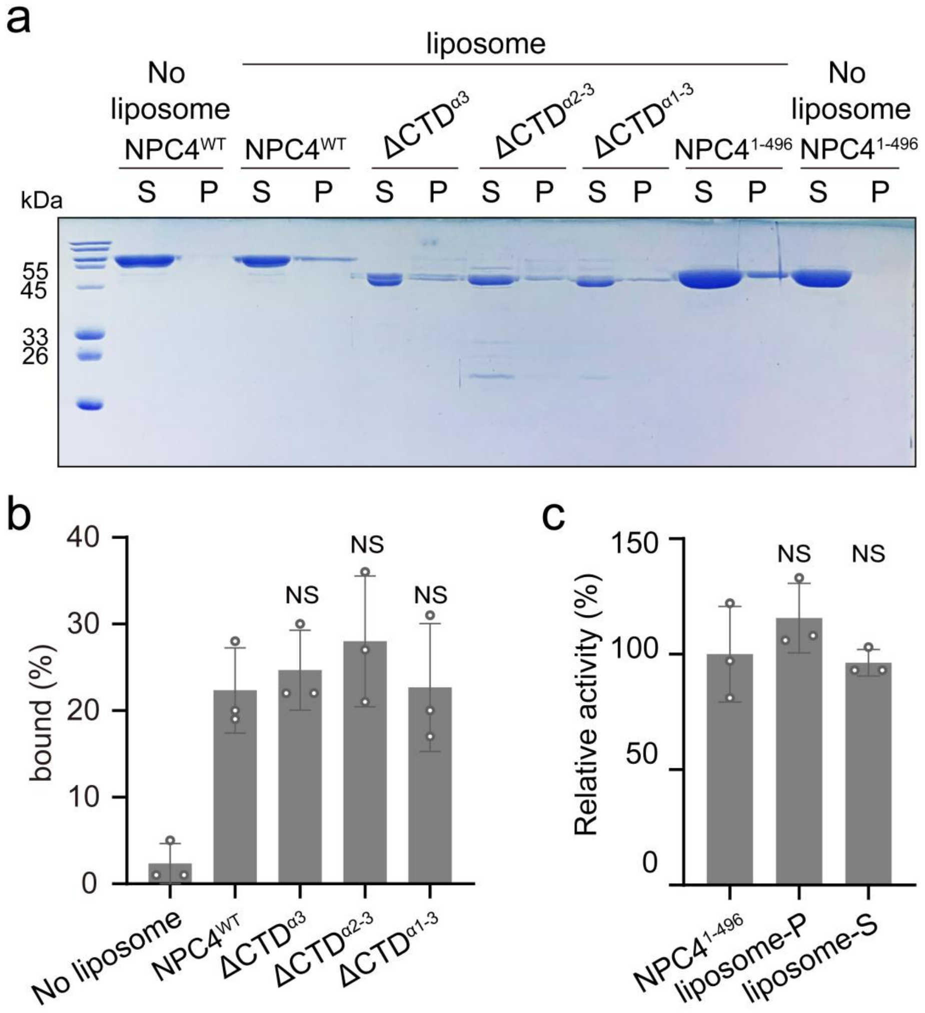

Supplementary Figure 14. NPC4-liposome association and the induced effect on enzyme activity by bilayer binding. **a**, Liposome sedimentation assay for protein-liposome association. Without liposome, no NPC4^{WT} or NPC4$^{1-496}$ presents in the pellet fraction after sedimentation, indicating that they are stable in the assay. By incubating with liposome, NPC4^{WT} (1-538), ΔCTD^{α3} (1-456), ΔCTD$^{α2-3}$ (1-443) and

$\Delta CTD^{\alpha 1\text{-}3}$ (1-415) bind liposome with similar strength, indicated by the presence of approximately equal amounts of protein in the pellet fraction. Thus, the deletion of $CTD^{\alpha 3}$, $CTD^{\alpha 2\text{-}3}$ or $CTD^{\alpha 1\text{-}3}$ should have little effect on NPC4-liposome association. We then incubated $NPC^{1\text{-}496}$ with the liposome, separately collected the pellet and supernatant fractions, and subjected them for activity assay. Source data are provided as a Source Data file. **b**, Quantify NPC4-liposome association based on the pellet and supernatant fractions resolved on SDS-PAGE gel of **a**. The data were collected from three independent experiments and were averaged, and the error indicates SD. Statistical analysis: unpaired two-tailed *t*-tests. NS, not significant. Source data are provided as a Source Data file. **c**, Activity assay. Liposome-P and liposome-S are the pellet and supernatant fractions of the incubated $NPC4^{\text{-}1496}$/liposome mixture, respectively, collected by sedimentation (see Methods for detail). The data from three independent measurements are averaged, and the error indicates SD. Statistical analysis: unpaired two-tailed *t*-tests. NS, not significant. Source data are provided as a Source Data file.

Supplementary Table1. Statistics of crystal data collection and structures refinement.

	**NPC4$^{1-415}$ (8HAV)**	**NPC4$^{1-496}$ (8HAW)**
**Data collection**		
Space group	$P\ 2_1\ 2_1\ 2_1$	$P\ 1\ 2_1\ 1$
Cell dimensions		
a, *b*, *c* (Å)	59.06 121.44 157.38	63.05 61.62 132.80
α, *β*, *γ* (°)	90.00 90.00 90.00	90.00 103.59 90.00
Resolution (Å)	50.00-2.10 (2.15-2.10)	50.00-2.10 (2.15-2.10)
No. reflections	860667 (59406)	387788 (30451)
R_{merge}	0.085 (0.421)	0.11 (0.257)
$I / \sigma I$	18.9 (5.8)	10.1 (5.8)
Completeness (%)	100.0 (100.0)	99.9 (100)
Redundancy	6.7	6.7
CC (1/2)	0.998 (0.969)	0.995 (0.986)
**Refinement**		
R_{work} / R_{free}	0.1886/0.2124	0.1738/0.2039
No. atoms		
Total	6986	8506
Protein	6407	7724
Ligand	0	2
Solvent molecules	579	780
B-factors		
Total	38.270	25.863
Protein	38.012	25.22
Ligand	0	14.565
Solvent molecules	41.115	32.194
R.m.s. deviations		
Bond lengths (Å)	0.003	0.006
Bond angles (°)	0.653	0.897
**Ramachandran Plot**		
Favored (%)	96.08	96.82
Allowed (%)	3.92	3.18
Outliers (%)	0	0

Statistics for the highest resolution shell are shown in parentheses.

Supplementary References

1. Robert, X. & Gouet, P. Deciphering key features in protein structures with the new ENDscript server. *Nucl Acids Res.* **42**, W320-324 (2014).
2. Gautier, R., Douguet, D., Antonny, B. & Drin, G. HELIQUEST: a web server to screen sequences with specific alpha-helical properties. *Bioinformatics* **24**, 2101–2102 (2008).
