# Supplementary material for: Insights into the mechanism of phospholipid hydrolysis by plant non-specific phospholipase C
Source: Nat Commun. 2023 Jan 12;14:194. doi: 10.1038/s41467-023-35915-4 (PMC9837106; doi:10.1038/s41467-023-35915-4)
Supplement: Supplementary file 3 — Description of Additional Supplementary Files [file 41467_2023_35915_MOESM3_ESM.pdf]

Description of Additional Supplementary Files

Supplementary Data 1: Primers used within this study.
